# The Missing Protein: Is T-Cadherin a Previously Unknown GPI-Anchored Receptor on Platelets?

**DOI:** 10.3390/membranes11030218

**Published:** 2021-03-19

**Authors:** Maria N. Balatskaya, Alexandra I. Baglay, Alexander V. Balatskiy

**Affiliations:** 1 Faculty of Medicine, Lomonosov Moscow State University, Lomonosovskiy av. 27-1, 119192 Moscow, Russia; baglay.alexandra@yandex.ru (A.I.B.); balatsky@fbm.msu.ru (A.V.B.); 2 Laboratory of Molecular Endocrinology, Institute of Experimental Cardiology National Medical Research Center of Cardiology, 3rd Cherepkovskaya st., 15a, 121552 Moscow, Russia

**Keywords:** platelets, T-cadherin, cadherin-13, GPI-anchored protein

## Abstract

The membrane of platelets contains at least one uncharacterized glycosylphosphatidylinositol (GPI)-anchored protein according to the literature. Moreover, there is not enough knowledge on the receptor of low-density lipoproteins (LDL) mediating rapid Ca^2+^ signaling in platelets. Coincidentally, expression of a GPI-anchored protein T-cadherin increases LDL-induced Ca^2+^ signaling in nucleated cells. Here we showed evidence that supports the hypothesis about the presence of T-cadherin on platelets. The presence of T-cadherin on the surface of platelets and megakaryocytes was proven using antibodies whose specificity was tested on several negative and positive control cells by flow cytometry and confocal microscopy. Using phosphatidylinositol-specific phospholipase C, the presence of glycosylphosphatidylinositol anchor in the platelet T-cadherin form as well as in other known forms was confirmed. We showed by immunoblotting that the significant part of T-cadherin was detected in specific membrane domains (detergent Triton X-114 resistant) and the molecular weight of this newly identified protein was greater than that of T-cadherin from nucleated cells. Nevertheless, polymerase chain reaction data confirmed only the presence of isoform-1 of T-cadherin in platelets and megakaryocytes, which was also present in nucleated cells. We observed the redistribution of this newly identified protein after the activation of platelets, but only further work may explain its functional importance. Thus, our data described T-cadherin with some post-translational modifications as a new GPI-anchored protein on human platelets.

## 1. Introduction

Platelets are small, non-nucleated blood components, challenging for research due to their structure and functions. Platelets are essential mediators of hemostasis, thrombosis, and vascular homeostasis, and their role in atherosclerosis and thrombotic events is well established. Since the global burden of cardiovascular diseases is increasing, platelet studies attract great attention.

Low-density lipoproteins (LDL) are considered one of the main inducers of atherogenesis; however, they not only undergo endocytosis and participate in foam cell formation, but also trigger intracellular signaling in endothelial and smooth muscle cells, as well as in platelets [1]. Platelets lack the “classical” apo-B,E, LDL receptor (LDLR) [2,3]. Until now, the receptor which induces the fast increase in intracellular concentration of inositol 1,4,5-triphosphate and Ca^2+^ after LDL binding to platelets has not been identified. A splice variant of the LDL receptor family member, apolipoprotein E receptor 2 (ApoER2’) is the most likely LDL receptor candidate on platelets. According to the results of Prof. Akkerman’s group, it induces phosphorylation of p38MAPK [4,5,6]. However, signaling from Apo ER2’ does not explain the key moment of platelet activation—direct fast Ca^2+^ mobilization [2,7,8,9,10,11]. Phosphoinositide metabolism and increase of intracellular Ca^2+^ occur very fast (several seconds), even when LDL concentrations are ten times lower than in works on Apo ER2’ [4,5,12]; thus, an unknown receptor may have a higher affinity for LDL. Other scientists believe that LDL binds to a G-protein coupled receptor based on the fact that LDL signaling is inhibited by the pertussis toxin [11].

We hypothesize that this unidentified receptor might be a glycosylphosphatidylinositol (GPI)-anchored protein T-cadherin (H-cadherin, cadherin-13). Moreover, it is known that there is a still uncharacterized GPI-anchored protein on platelets [13,14]. Earlier, it was discovered that T-cadherin induces phosphoinositide and calcium signaling in smooth muscle cells and T-cadherin expressing cells upon binding with LDL [15,16,17], and this signaling is very similar to the activation observed in platelets [2,8,9,18,19,20]. Several other GPI-anchored proteins can induce phosphoinositide and calcium signaling [21,22,23,24,25,26,27]. It was shown that these proteins directly bind to Gαi [28,29], a specific substrate of the pertussis toxin, which explains its effects on platelets [11]. However, we do not know enough about signaling of GPI-anchored proteins, especially in platelets. Membrane cholesterol-enriched domains, rafts, seem to play an important role in signal transduction in platelets [30]. We have previously shown that LDL induce the formation of short-lived T-cadherin clusters and demonstrated the cholesterol-dependence of calcium signaling [31]. Another ligand of T-cadherin, adiponectin (adipocyte-derived hormone), induces long-lived T-cadherin clusters, cholesterol-independent internalization of T-cadherin, and enhances exosome biogenesis [31,32]. Binding of T-cadherin and adiponectin is essential for beneficial effects in the cardiovascular system [33,34,35,36], but no information about this binding on platelets has been published.

Here, we presented evidence suggesting that GPI-anchored T-cadherin is indeed one of the previously unknown proteins on platelets.

## 2. Materials and Methods

### 2.1. Cell Culture

The MEG-01 (human megakaryoblastic cell line, ATCC) and THP-01 (human monocytic cell line from the collection of Moscow State University https://human.depo.msu.ru, accessed on 3 September 2018) cell lines were cultured in RPMI-1640 complete growth medium (Thermo Fisher, Carlsbad, CA, USA) containing an antibiotic-antimycotic (HyClone, Marlborough, MA, USA) and 10% fetal bovine serum (FBS, HyClone). Primary human umbilical vein endothelial cells (HUVEC, https://human.depo.msu.ru, accessed on 3 September 2018) were cultured in EBM-2 complete medium with additives (Lonza, Basel, Switzerland), an antibiotic-antimycotic (HyClone), and 2% FBS (HyClone). Chinese hamster ovary (CHO) cells were cultured in Dulbecco’s modified Eagle’s medium-F12 (HyClone or PanEco) with an antibiotic-antimycotic solution (HyClone) and 10% FBS (HyClone). All cells were passaged by treatment with the Versene solution (PanEco, Moscow, Russia) and HyQTase protease mixture (HyClone). All cell lines were cultured in an incubator at 37 °C and 5% CO_2_.

### 2.2. Isolation of Platelets

Platelets were isolated from the blood of healthy volunteers collected for the biobank of Moscow State University (https://human.depo.msu.ru, accessed on 3 September 2018). The procedures were approved by the Local Ethics Committee of the Medical Scientific and Educational Center, Lomonosov Moscow State University (protocol No.4 from 4 June 2018). The blood was mixed with a 3.8% sodium citrate solution in a blood:anticoagulant ratio of 9:1 and centrifuged immediately after collection for 15 min at 150× *g* and room temperature (RT). Platelets washed from most of the blood proteins were obtained by centrifugation with citrate buffer or gel filtration on a column with Sepharose CL-2B according to the method described by Krueger and colleagues [37]. Washed platelets were resuspended in modified HEPES/Tyrode’s buffer (10 mM HEPES, 137 mM NaCl, 2.8 mM KCl, 1 mM MgCl_2_, 12 mM NaHCO_3_, 0.4 mM Na_2_HPO_4_, 0.35% (*w*/*v*) BSA, 5.5 mM glucose, pH 7.4), and resting platelets were resuspended in the buffer with inhibitors of platelet aggregation (50 ng/mL prostaglandin E1).

### 2.3. Flow Cytometry

Four types of cells were used: Washed platelets, MEG-01, THP-1, and HUVEC. HUVEC were detached from the plates using the Versene solution (PanEco) and a mixture of proteases HyQTase (HyClone). Detached HUVEC, mechanically removed MEG-01, and suspended THP-1 cells were centrifuged at 150× *g* for 10 min. All line cells were resuspended in Hank’s solution with 10 mM HEPES and 0.35% BSA. One half of the cells and platelets was incubated with biotinylated anti-T-cadherin antibody (R&D, Minneapolis, MN, USA, #BAF3264), and the other half was incubated with isotype control (R&D #BAF108) for 30 min at RT. Then we added Dylight 649-streptavidin (Jackson ImmunoResearch, Ely, UK, #016-490-084), FITC anti-human CD61/integrin beta-3 (clone VI-PL2, Biolegend, San Diego, CA, USA, #336404), or FITC mouse IgG1, κ isotype control antibody (clone MOPC-21, Biolegend #400110) for 30 min at RT. After washing, the cells were resuspended in 400 μL of modified HEPES/Tyrode’s buffer and measured using a FACSCanto II flow cytometer (BD Biosciences, Franklin Lakes, NJ, USA). For some experiments we used primary anti-T-cadherin antibodies (ProSci, Poway, CA, USA, #3583, Santa Cruz, Dallas, TX, USA, #7940, Abnova, Taipei City, Taiwan #H0001012-001), control normal rabbit IgG (ProSci #3703), and secondary Alexa Fluor 647 AffiniPure F(ab’)_2_ fragment donkey anti-rabbit IgG (H + L) (Jackson ImmunoResearch #711-606-152).

Data analysis was performed using the FlowJo software (BD Biosciences). The Kruskal–Wallis test was used for statistical analysis of flow cytometry data.

### 2.4. Confocal Microscopy

CHO cells were transfected with plasmids pIRES-T-cad [31,38] using lipofectamine 2000 (Invitrogen, Waltham, MA, USA) in accordance with the manufacturer’s protocol and fixed in 4% paraformaldehyde solution (PFA, Sigma–Aldrich, Munich, Germany) for 15 min at RT. For immunostaining we used biotinylated anti-T-cadherin antibodies (R&D #BAF3264) or isotype control (R&D #BAF108) for an hour, Dylight 649-streptavidin (Jackson ImmunoResearch #016-490-084) for an hour, and Hoechst 33342 (Life Technologies, Waltham, MA, USA, #H3570) for 15 min to detect DNA.

The suspension of live washed platelets was incubated with anti-CD61 antibodies, anti-T-cadherin antibodies, and isotype control as described above. In the experiment with nucleated blood cells, Hoechst 33342 was used for 15 min. After incubation with antibodies, the cells were washed in Tyrode’s buffer, and then live cells were seeded on an 8-well slide chamber (Nunc Lab-Tek, Thermo Fisher Scientific, Waltham, MA, USA). Live platelets were adhered and activated on the glass surface.

To obtain an image of resting platelets, the cells were also incubated with antibodies after fixating for 15 min in 1% PFA at RT according to [37]. Some cells were fixed and permeabilized using the perm/wash solution containing saponin (BD Biosciences) in accordance with the manufacturer’s protocol.

Visualization and analysis were performed using a laser scanning confocal microscope LSM 780 (Zeiss, Oberkochen, Germany) equipped with Plan-Apochromat 63x lens (1.4 numerical aperture) and Zen 2010 software (Zeiss) or ImageJ software (NIH). We used 405, 488, and 633 nm lasers for excitation Hoechst 33342, FITC, and DyLight649, respectively.

For colocalization analysis, we used the Coloc 2 plugin in ImageJ. We selected the regions of interest (platelets) and calculated Pearson’s R value above automatic threshold.

### 2.5. Phospholipase Digestion

For GPI-anchor digestion we used phosphatidylinositol-specific phospholipase C (PI-PLC) from *Bacillus cereus* (Invitrogen, Waltham, MA, USA, #P6466). According to the manufacture’s protocol, this enzyme may be used at 4 °C as at 37 °C. To optimize the temperature, we tested the activity of PI-PLC at 4 °C, RT, and 37 °C using peripheral blood mononuclear cells (PBMC) and checked GPI-anchored CD14 on their surface by flow cytometry (data not shown). The best result was observed at 4 °C, a similar result was obtained at RT, and minimum changes were found at 37 °C. For experiments with platelets, we tested two temperatures (RT and 37 °C), because the cold activation of platelets at 4 °C is known. We demonstrated that RT was more suitable and used it later. Thus, washed platelets were incubated for 90 min at RT with 2 unit/mL PI-PLC and washed before analysis.

### 2.6. Polyacrylamide Gel Electrophoresis of Proteins, Immunoblotting, and Immunoprecipitation

The washed platelets were lysed using Laemmli sample buffer with β-mercaptoethanol (Sigma-Aldrich). HUVEC were lysed in the same way or as described previously [31]. Samples were denatured at 95 °C for 5 min.

Immunoprecipitated samples from platelets were prepared as described in [39]. Briefly, we used Dynabeads M-280 Streptavidin (Invitrogen) and biotinylated antibody anti-T-cadherin (R&D #BAF3264).

Proteins were separated by 4–15% sodium dodecyl sulfate polyacrylamide gel electrophoresis or in AnykD TGX stain-free precast gel. Proteins were transferred to a polyvinylidene fluoride membrane (GE Healthcare, Chicago, IL, USA) for 2 h at 350 mA at 4 °C. Blocking of nonspecific binding to the membrane was performed using 5% nonfat milk in PBS with 0.05% Tween 20 overnight at 4 °C. Membranes were incubated with primary polyclonal rabbit (ProSci #3583) or goat (R&D, #AF3264) antibodies against T-cadherin overnight at 4 °C or for 1 h at RT. Secondary antibodies against rabbit or goat IgG, conjugated with horseradish peroxidase (Sigma, SAB3700852; IMTEK P-GAR Iss; R&D, #HAF109) were used for 1 h at RT. An enhanced chemiluminescent horseradish peroxidase substrates Pico or Dura (Pierce) were used to visualize the proteins on the membrane. The luminescence was recorded on a ChemiDoc instrument (Bio-Rad, Hercules, CA, USA) or was detected with X-ray film (Kodak, New York, NY, USA). For image analysis, Quantity One or Image Lab (Bio-Rad) was used.

### 2.7. Polymerase Chain Reaction and Sanger Sequencing

For sequences of T-cadherin transcript variants 1, 2, 3, 4, 5, and 6, we used NCBI Reference Sequences NM_001257.5, NM_001220488.2, NM_001220489.2, NM_001220490.2, NM_001220491.2, and NM_001220492.2 accordingly. RNA from HUVEC and MEG-01 cells was isolated using the RNeasy Mini kit (Qiagen, Hilden, Germany) in accordance with the manufacturer’s protocol. To isolate RNA from platelets, EDTA-stabilized blood was centrifuged at 100× *g* without braking for 20 min in order to obtain platelet-rich plasma. Then 50 mL of this plasma was centrifuged at 3000× *g* for 15 min in order to pellet the platelets. The supernatant was removed, while the pellet was resuspended in 20 mL of PBS and passed through a filter (Sigma–Aldrich, Millex, #F8023) with a pore diameter of 5 μm to remove the leukocytes. The filtrate was centrifuged at 3000× *g* for 10 min. Resuspension in PBS and centrifugation was repeated one more time. Then 6 mL of the ExtractRNA reagent (Evrogen, Moscow, Russia) was added to the pellet. The further process of RNA isolation was carried out in accordance with the manufacturer’s protocol. Before precipitation of RNA, the co-precipitant Satellite Red (Evrogen) was added to the solution.

Reverse transcription was performed using the MMLV RT kit (Evrogen) with oligo(dT) primers in accordance with the manufacturer’s protocol. For PCR, 4 μL of the reverse transcription reaction mixture was added to the reaction, the final volume was 50 μL. The following primers at a concentration of 0.4 μM and the annealing temperature of 58 °C were used:

Pair 1:

Forward: CDH13_trscr_CDS1_F (3′-AAAATGCAGCCGAGAACTCC-5′), reverse: CDH13_trscr_CDS4_R (5ʹ-CCCCCGACAATCACGAGTTC-3′).

Product length: 344 bp for transcript variants 1, 5, 6; 450 bp for transcript variant 2.

Pair 2:

Forward: T-cad-PP-for1_CDS3 (3′-GGGGAAAGACATCCAGGGCTCC-5′), reverse: T-cad-EC1-rev1_CDS6 (5ʹ-TCGAGAGTTTTGCCATTGACATCAG-3′).

Product length: 339 bp for transcript variants 1, 2; 222 bp for transcript variant 3.

Pair 3:

Forward: CDH13_trscr_CDS6_F (3′-ATGTCAATGGCAAAACTCTCGA-5′), reverse: CDH13_trscr-seq_CDS9_R (5ʹ-TGGGGTCATCCTTATCTTCAACTGTCA-3′).

Product length: 519 bp for transcript variants 1, 2, 3, 4.

Pair 4:

Forward: CDH13_trscr_CDS8_F (3′-TCGATGACAAAAATGATCACTCACCAAA-5′), reverse: CDH13_trscr-seq_CDS14_R (5ʹ-ACGTCAGGAGTTCTCACAGACAA-3′).

Product length: 1098 bp for transcript variants 1, 2, 3, 4.

Besides this, two more primers were designed to sequence the fragment from pair 4: forward CDH13_trscr-seq_CDS11_F (3′-CCGTGAGTCCCCATTTGTCGACA-5′) and reverse CDH13_trscr-seq_ex11_R (3′-GCCACGTAG).

PCR products were isolated from the gel using a QIAquick Gel Extraction kit (Qiagen). The resulting products were additionally precipitated with ethanol in the presence of 80 mM sodium acetate.

Sanger sequencing was performed using the BigDye Terminator v1.1 Cycle Sequencing kit (Thermo Fisher Scientific, Waltham, MA, USA) in accordance with the manufacturer’s protocol. Purification of the sequencing products was performed using the BigDye XTerminator Purification kit (Thermo Fisher Scientific) in accordance with the manufacturer’s recommendations. For capillary electrophoresis, a 3730xl DNA Analyzer sequencer (Thermo Fisher Scientific) was used.

### 2.8. Isolation of Detergent Insoluble Fractions from Platelets

Resting washed platelets were lysed in cold lysis buffer with Triton X-114 and membrane raft fractions were isolated using gradient ultracentrifugation in sucrose of various densities in accordance with the protocol from Shrimpton et al. [40].

After ultracentrifugation, the gradient was divided into 12 fractions of 830 μL each. Proteins from all 12 fractions were precipitated with trichloroacetic acid, dissolved in sample buffer with dithiothreitol (Sigma) and analyzed for the presence of T-cadherin using immunoblotting. The total amount of protein in each fraction was estimated using amide black (Fisher BioReagents, Thermo Fisher Scientific).

## 3. Results

### 3.1. Specific Epitope of T-Cadherin Identified on Membranes of Human Platelets and Megakaryocytes

**Figure 1:**
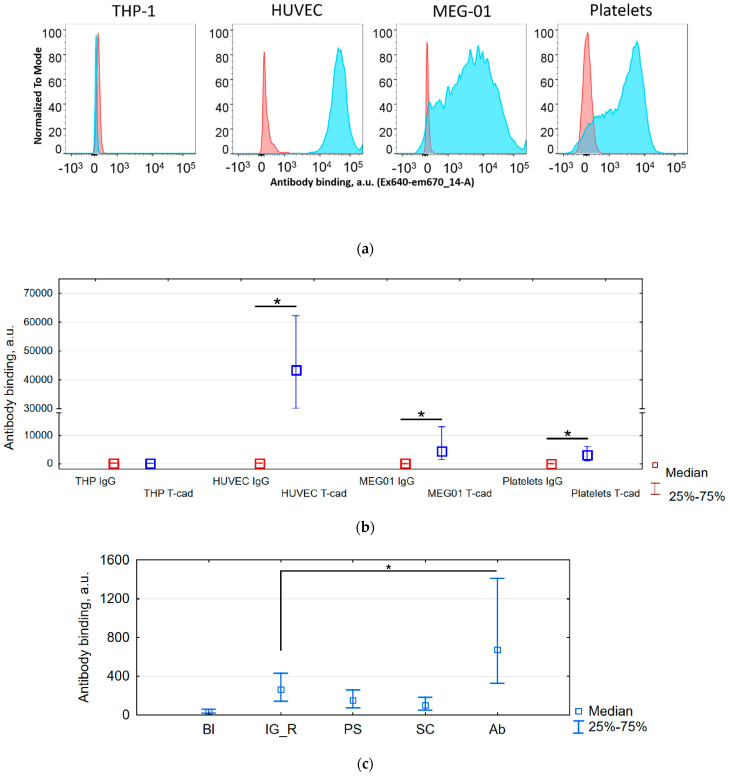
Specific binding of anti-T-cadherin antibodies to megakaryocytes MEG-01 and platelets compared with negative THP-1 control and positive human umbilical vein endothelial cells (HUVEC) control according to flow cytometry data. Forward scatter /Side scatter gates were cell specific and channel voltage for detection of T-cadherin (ex640 em670/14) was the same for all cells (THP-1, HUVEC, MEG-01) and different for platelets. (**a**) Distribution histograms of cells incubated with antibodies anti-T-cadherin (blue) or isotypic control (red). (**b**) Median and percentiles of the fluorescence (antibody binding) for the above histograms. * The binding of the specific antibody is greater than of the isotype control, *p* < 0.001. *n* = 5. (**c**) Median and percentiles of the fluorescence (antibody binding) for blank sample without antibody (Bl), sample with rabbit IgG (IG_R), ProSci (PS), SantaCruz (SC), and Abnova (Ab) antibodies. * The binding of the specific antibody is greater than of the isotype control, *p* < 0.001. *n* = 2.

We checked the presence of T-cadherin epitopes on the surface of human platelets and megakaryocytes using a specific antibody (R&D, BAF3264) and flow cytometry. The anti-T-cadherin antibody precisely bound with HUVEC (positive control, cells expressing T-cadherin), MEG-01 (a megakaryoblastic cell line), and platelets, but did not bind with THP-1 (negative control, cells which do not express T-cadherin according to proteinatlas.org and [41]). We demonstrated a significant difference in binding (*p* < 0.001, pairwise comparison, Figure 1a,b) between the specific antibody and the corresponding isotype control in all types of cells. In samples of HUVEC, MEG-01, and platelets, we detected specific binding greater than with the isotype control. In the sample of THP-1 we did not detect any binding and the signal from the anti-T-cadherin antibody was less than from the isotype control. Such an effect was described by Andersen et al. [42]; thus, using an isotype control instead of non-stained control was a stricter condition for obtaining positive results. It is interesting to note that other antibodies (ProSci, 3583; Santa Cruz 7940) against the first domains of T-cadherin were hardly bound with platelets at all, and the signal was no higher than that from the isotype control (Figure 1c).

**Figure 2:**
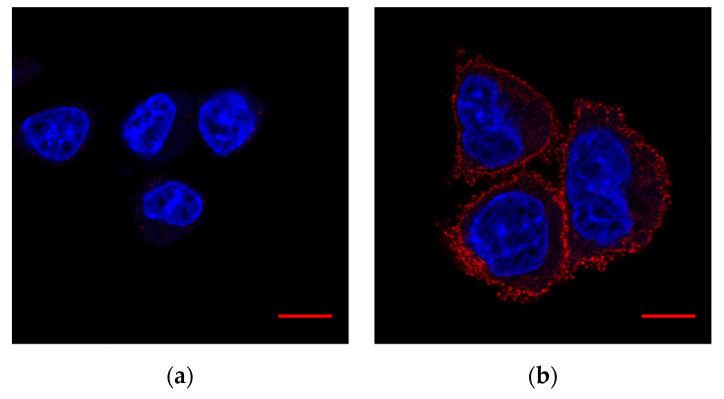
Validation of antibody #BAF3264. Fixed cells. Hoechst 33342 (DNA)—blue, #BAF3264 (T-cadherin)—red: (**a**) Wild-type CHO; (**b**) CHO cells with T-cadherin expression. Scale bar (red line) 10 µm.

For an additional estimate of potential cross-reactivity with other proteins we tested the same antibodies with wild-type CHO (cells without T-cadherin endogenous expression according to [43]) and CHO cells transfected by a plasmid with T-cadherin cDNA (Figure 2). Such positive and negative controls confirmed the specificity of the antibody.

**Figure 3:**
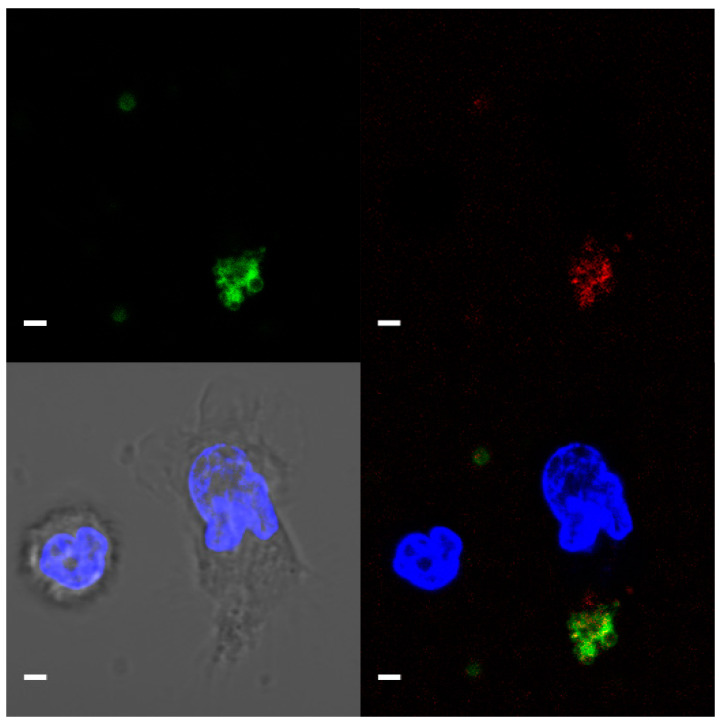
Representative confocal microscopy image (a single focal plane) of T-cadherin on platelets. CD61—green, T-cadherin—red, DNA (Hoecst 33342)—blue. Live platelet and nucleated blood cells. Scale bar (white line) 2 µm.

T-cadherin was detected on the surface of platelets but not on leukocytes using immunostaining and confocal microscopy (Figure 3).

Thus, the antibody BAF3264 did not cross-react with the surface of THP-1, CHO, leukocytes, and specifically bound with platelets, MEG-01, and HUVEC.

### 3.2. T-Cadherin on Platelets and Megakaryocytes Is GPI-Anchored

**Figure 4:**
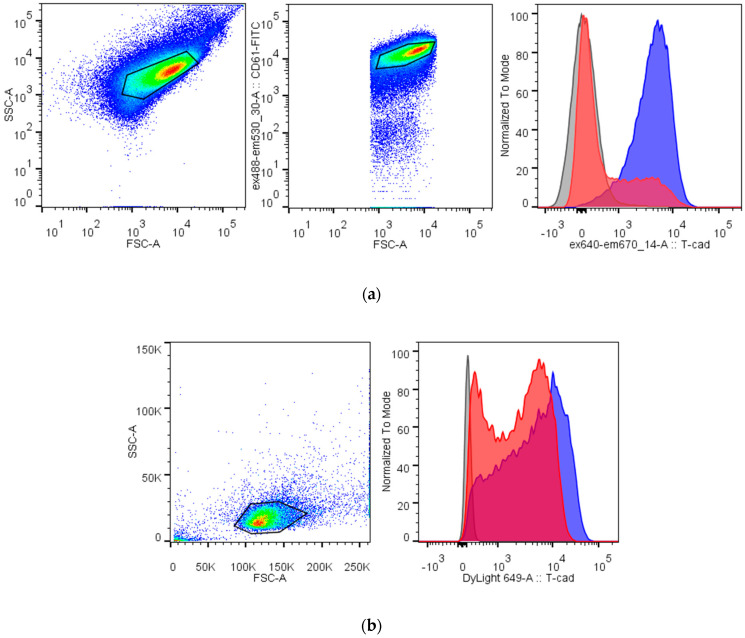
Digestion with phosphatidylinositol-specific phospholipase C (PI-PLC) decreases binding of the specific antibody against T-cadherin to platelets (**a**) and MEG-01 (**b**). Platelets are identified by their FSC/SSC characteristic (the first gate) and positive CD61 labeling (the second gate), MEG-01 cells are identified by the other FSC/SSC gate. Right histograms show fluorescence from the binding of the anti-T-cadherin antibody before (blue) and after (red) PI-PLC treatment and from the isotypic control (gray).

The important structure feature of T-cadherin in nucleated cells is the GPI-anchor. To test whether the T-cadherin form on platelets and megakaryocytes is GPI-anchored, we treated cells with a specific phospholipase PI-PLC. Antibody (R&D, BAF3264) binding with platelets and MEG-01 decreased after PI-PLC digestion (Figure 4). In both cases, we noticed two populations of platelets or MEG-01 after PI-PLC treatment.

We detected a reliable signal from the other antibody against full-length T-cadherin (Abnova, H0001012-001) after binding with platelets and its decrease after PI-PLC treatment too (data not shown).

Thus, at least some of the T-cadherin protein on platelets and megakaryocytes exists in a GPI-anchored form.

### 3.3. Detection of Unusual T-Cadherin Molecular Weight in Megakaryocytes and Platelets

**Figure 5:**
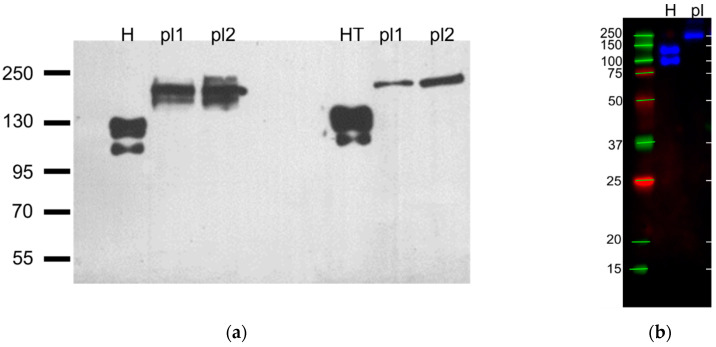
Representative immunoblots against T-cadherin with platelet and positive controls lysates with protein bands standards, *n* = 5. (**a**) First three lanes were incubated with R&D #AF3264 antibodies, last three lanes were incubated with ProSci #3583 antibodies, H–HUVEC, pl1, pl2—platelets from different donors, HT–HEK293 overexpressing T-cadherin (preparation describe in [31]) (film), (**b**) H–HUVEC, pl—immunoprecipitate of the platelet lysate (R&D #AF3264, multichannel digital detection).

To estimate the molecular weight of T-cadherin on platelets we made an immunoblot with different antibodies. We used lysates of HUVEC and HEK293 overexpressing T-cadherin as a positive control (Figure 5).

In the HUVEC, HEK293-T-cad, and CHO-T-cad ([44]) samples, we detected two bands: The T-cadherin precursor (130 kDa) and the mature (105 kDa) form [45], while in platelet lysates we identified a T-cadherin epitope of another molecular mass. It was greater than 160 kDa in all blots. In Figure 5a it was about 200 kDa, and in Figure 5b about 250 kDa similar to the heaviest standard band.

To figure out the reason for such heavy molecular weight and to confirm the results by an independent method we tried to amplify the mRNA of the T-cadherin gene (CDH13).

### 3.4. Isoform-1 of T-Cadherin Is Found in Platelets and Megakaryocytes

**Figure 6:**
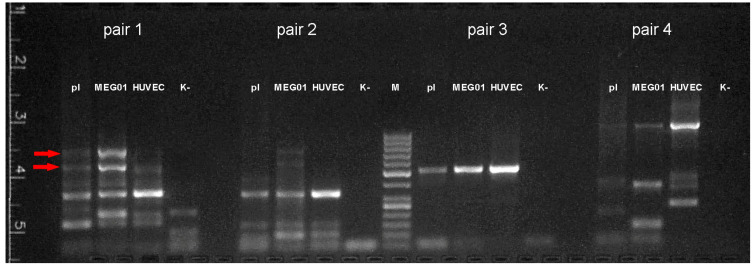
Results of reverse transcription PCR with RNA isolated from platelets (pl), MEG01, and HUVEC cells. M—size marker (50, 100, 150, 200, 250, 300, 400, 500, 600, 700, 800, 900, 1000 bp), K-—negative control. Arrows indicate fragments missing or poorly represented in HUVEC.

The low concentration of RNA in platelets requires suboptimal PCR conditions leading to the formation of many nonspecific annealing products. Nevertheless, our data demonstrate that transcript variant 1 of T-cadherin is present in all RNA samples (platelets, MEG-01, and HUVEC) since the 450 bp PCR product corresponding to transcript variant 2 when using the primer pair #1 is practically undetectable (Figure 6).

To identify a potentially existing long transcript variant of T-cadherin mRNA, all PCR products absent in the negative control (i.e., not primer-dimers) were isolated from the agarose gel. Special attention was paid to the two bright high-molecular-weight PCR products of pair #1, which are practically absent in cDNA samples from HUVEC but are present in cDNA samples from platelets and especially from MEG-01. These products were about 550 and 800–900 bp in length.

All products were sequenced, but we were unable to obtain a signal during sequencing of high molecular weight (the 550 and 800–900 bp) PCR products from primer pair #1. Apparently, these products result from nonspecific annealing of primers to cDNA of some transcripts present in platelets and megakaryocytes, but absent in HUVEC. At the same time, we succeeded in sequencing the other PCR products of primer pair 1 (344 bp, partially), pair # 2 (339 bp), pair # 3 (519 bp), and pair # 4 (1098 bp, partially). The order of exons in the resulting sequence corresponds to T-cadherin splice variant 1.

Thus, according to our data, only isoform-1 of T-cadherin mRNA is present in platelets and megakaryocytes.

### 3.5. Redistribution of T-Cadherin after Platelets Activation

**Figure 7:**
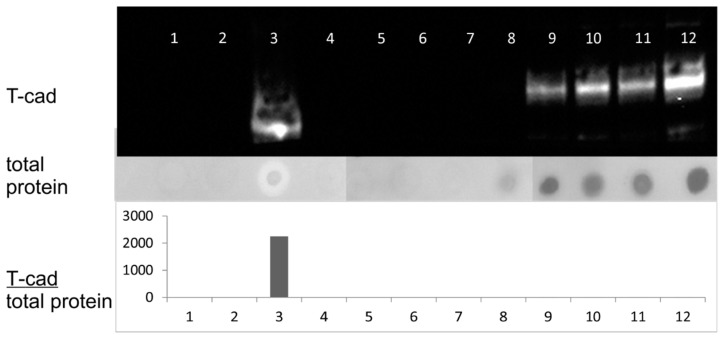
Analysis of fractions after ultracentrifugation of platelet lysates with the Triton X-114 detergent. 1–12—the fractions obtained after ultracentrifugation. Upper panel—immunoblotting of the obtained fractions with antibodies against T-cadherin. The middle panel shows the total amount of protein in the fractions, analyzed using amide black. The lower panel shows the ratio of the T-cadherin signal intensity measured by immunoblotting to the total protein amount in the fractions, arbitrary units.

Previously we have demonstrated that the functional activity of T-cadherin depends on its oligomeric structure and the composition of the membrane with which it is associated [31]. Here we checked different membrane fractions of platelets and figured out that the significant part (26%) of the protein of interest is located in the detergent resistant fraction (Figure 7). This suggests that T-cadherin is heterogeneously distributed on the membrane of platelets, possibly in the cholesterol-enriched membrane microdomains, rafts, which are Triton-X114 resistant.

**Figure 8:**
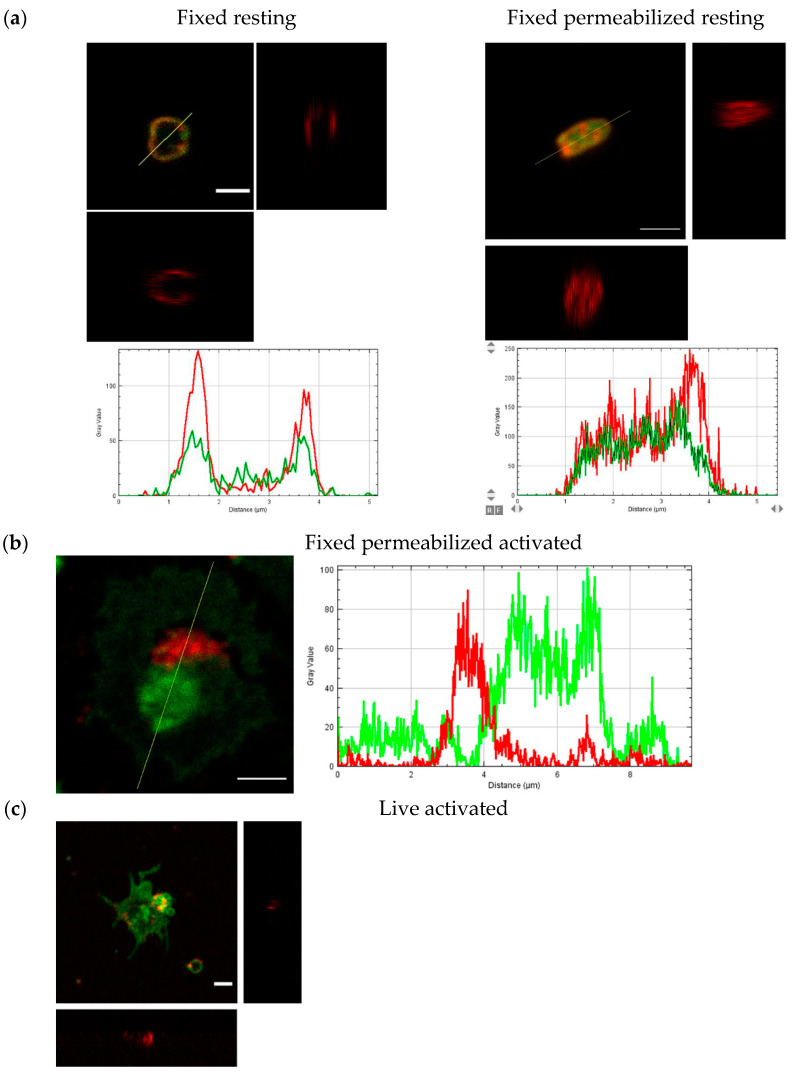
The confocal microscopy images and orthogonal view of Z-stack of resting and activated platelets. CD61—green, T-cadherin—red. Plots of fluorescence intensity along the cross-section line from (**a**,**b**), respectively. (**a**) Fixed resting human platelets—non-permeabilized (left) and permeabilized (right); (**b**) fixed and permeabilized activated human platelet; (**c**) live activated human platelet. Scale bar (white line) 2 µm.

We detected localization of T-cadherin on the membrane of resting platelets (Figure 8a, Pearson’s R value is 0.67 for fluorescence intensities of T-cadherin and CD61 channels) by confocal microscopy too. Some part of T-cadherin was detected inside the platelets (Figure 8b, Pearson’s R value is 0.51). We showed the redistribution of T-cadherin after platelet activation—a reverse correlation with localization of CD61 was demonstrated (Figure 8c Pearson’s R value is −0.28).

Thus, T-cadherin was detected on the surface of resting platelets in the detergent resistant membrane fraction and changed the localization after activation.

## 4. Discussion

The presence of T-cadherin on the platelet membrane is supported by (1) the specific binding of antibodies to platelets and megakaryocytes, detected by flow cytometry, confocal microscopy, and immunoblotting, (2) a decrease of this binding after phospholipase treatment, and (3) detection of T-cadherin mRNA in platelets and MEG-01. In the future, it will be interesting to obtain more information about this protein on platelets by different approaches. Several of our attempts to use mass spectrometry in order to determine the primary sequence of this protein failed. Possibly due to the potentially high degree of glycosylation of the protein of interest and its hydrophobic nature. We detected several peptides of other cadherins, some of which as far as we know from literature can be found in platelets: E-cadherin (cadherin-1), N-cadherin (cadherin-2), cadherin-4, cadherin-6, protocadherin [46,47,48,49,50], and some others according to the PlateletWeb data resource. Interestingly, Elrod and colleagues revealed a heavy band (>120 kDa) on the immunoblot using a pan-cadherin antibody.

Our findings can fill the gap of knowledge about GPI-anchored proteins on platelets. Polgar and colleagues found a heavily glycosylated 500 kDa GPI-anchored glycoprotein [13] but it has not been characterized yet [14]. In our blots the apparent molecular weight of the protein of interest was about 160–250 kDa. Glyco- and lipoproteins are usually not fully coated with SDS and do not behave as expected in SDS-PAGE, leading to inaccurate molecular weight estimations. High accuracy in protein molecular weight measurement has never been achieved with SDS-PAGE and prestained protein molecular weight markers [51,52]. Another explanation for the differences in the molecular weight may be in the difficulties with denaturation of membrane proteins or existence of other splice variants and glycoforms. We noticed that two different antibodies against full-length T-cadherin bound with the surface of platelets while the other two antibodies against its first two domains did not (according to flow cytometry data). Previously, we showed on HUVEC and HEK293-T-cad cells that different antibodies against T-cadherin recognized a different pattern (according to confocal microscopy data). Possibly, the N-terminal part of T-cadherin on platelets has some other modifications. This is important to keep in mind because these antibodies are commercially available and their usage for the detection of T-cadherin in blood can lead to different result [53,54]. Philippova and colleagues believe that platelet microparticles from human plasma were negative for T-cadherin [55]. Our data showed that platelet microparticles were positive for T-cadherin (Figure 8 and [56]). Recently, it was shown that T-cadherin (cadherin-13) content in platelet-derived microparticles changes in acute phase of antineutrophil cytoplasmic antibody (ANCA)-associated vasculitis [57]. In addition, it was demonstrated that T-cadherin plasma levels decrease in patients with gray platelet syndrome [58]. Moreover, the significant association between T-cadherin and both platelet count and mean platelet volume was discovered [59]. These facts support the role of T-cadherin in platelet biology.

To investigate the primary structure of the protein we used PCR and Sanger sequencing. We found the isoform-1 of T-cadherin mRNA in platelets and MEG-01. This provided evidence of T-cadherin synthesis in precursor cells, megakaryocytes. The calculated molecular weight of T-cadherin (isoform-1) according to the amino acid sequence was about 78 kDa. The observed molecular weight of T-cadherin in nucleated cells was 105 (mature) and 130 kDa (precursor), because this protein is highly glycosylated in these cells; thus, possibly in platelets, the degree of glycosylation might be even higher. Another possible reason is the stable dimers/complexes of T-cadherin, for example, are linked by transglutaminases.

According to our flow cytometry results, at least part of the protein of interest on the membrane is GPI-anchored. We demonstrated the effect of PI-PLC treatment but some populations of platelets and MEG-01 saved T-cadherin even after treatment. We assume that this may be due to re-exposure during labeling with antibodies, inaccessibility of the digest site for the enzyme, or the existence of another form of T-cadherin. It is interesting that in an experiment with protein CDw109 on human platelets, Smith and colleagues showed partial digestion too [60]. It is possible that there are two different phosphatidylinositol moieties of the GPI anchors: PI-PLC-sensitive and PI-PLC -resistant, as was found in case with CD52 [61]. We also cannot exclude platelet activation after PLC treatment and the redistribution of T-cadherin.

A substantial portion of platelet T-cadherin is found in the Triton X-114-resistant membrane fraction (and in the Triton X-100 resistant membrane fraction, data not shown), which is usually called membrane rafts. These findings suggest the important role of the membrane for the functional activity of GPI-anchored proteins. Previously, we demonstrated the clusterization of T-cadherin upon binding with ligands and the role of cholesterol and actin in the signal transduction in model cells [31]. Another GPI-anchored protein, CD55, is not predominantly distributed in the detergent-resistant membranes of resting platelets but the authors discussed the dependence of its localization within rafts from the clustering too [62].

In the present work, we showed the distribution of T-cadherin before and after activation using confocal microscopy. T-cadherin is partly located on the plasma membrane but some of it located inside the platelets, probably in the network of numerous invaginations and granules. After platelet activation on glass T-cadherin was not homogeneously distributed on the platelet surface, it was found in vesicles (Figure 8c and [56]) and in the open canalicular system (Figure 8b). Further studies are needed to evaluate the role of T-cadherin in platelet activation.

Some previous observations can be explained if T-cadherin is the LDL receptor on platelets. The question about what protein is the receptor of LDL which can induce rapid (sec-min) calcium signaling was discussed earlier [2,3,18]. Moreover, LDL increased the agonists-induced (thrombin, ADP, and epinephrine) rise of Ca^2+^ [8,11]. As was mentioned above, T-cadherin binds LDL and induces calcium signaling with the same dynamics in nucleated cells [8,9,16,20,63,64].

T-cadherin can bind another ligand–high molecular weight adiponectin which stimulates exosome biogenesis [32,65]. It is known that adiponectin causes anti-atherogenic effects, in some way opposite to the effects caused by LDL. We believe that LDL and adiponectin compete for binding to T-cadherin and the balance between them is important for the development of cardiovascular diseases.

This research has thrown up many questions in need of further investigations. These questions include the role of T-cadherin on platelets in Ca^2+^ mobilization upon binding with its ligands, its role in vesicle formation, hemostasis, and the development of cardiovascular diseases.

## Data Availability

The data presented in this study are available on request from the corresponding author.
